# Nodular Lymphangitis (Sporotrichoid Lymphocutaneous Infections). Clues to Differential Diagnosis

**DOI:** 10.3390/jof4020056

**Published:** 2018-05-09

**Authors:** Andrés Tirado-Sánchez, Alexandro Bonifaz

**Affiliations:** 1 Dermatology Service & Mycology Department, Hospital General de Mexico, “Dr. Eduardo Liceaga”, Balmis 148, Colonia Doctores, Ciudad de México 06726, Mexico; atsdermahgm@gmail.com; 2 Internal Medicine Department, Hospital General de Zona 29, Instituto Mexicano del Seguro Social, Ciudad de Mexico 07950, Mexico

**Keywords:** nodular lymphangitis, sporotrichoid lymphocutaneous infections, *Sporothrix schenckii*, diagnosis, treatment

## Abstract

Nodular lymphangitis, also known as sporotrichoid lymphocutaneous infections, is characterized by suppurative inflammatory nodules along the lymphatic vessels. This manifestation is classic of sporotrichosis, however, other infections such as nocardiosis, atypical mycobacteriosis, leishmaniasis, among others, can also express this clinical pattern. Sporotrichosis, which often occurs in gardeners, remains the most recognized cause of nodular lymphangitis. The histopathological studies, as well as the culture are diagnostic standards of lesions that do not respond to empirical treatment. In this article, we will review the main causes of nodular lymphangitis or lymphocutaneous sporotrichoid infections.

## 1. Introduction

There are many diseases that have the characteristic of following the lymphatic path, as in lymphangitis associated with group A streptococcal-infections, where the initial lesion extends rapidly from the inoculum zone to the regional lymph nodes [1]. A more severe and chronic form of lymphangitis presents with subcutaneous inflammatory nodules that follow the direction of the affected limb with a frequently indolent course [2,3]. The diagnosis of nodular lymphangitis, also known as sporotrichoid lymphocutaneous infections, indicates a group of infectious diseases with different causes (Table 1) as well as clinical, prognostic, and treatment characteristics (Table 2). Most cases of nodular lymphangitis are the result of infections and often develop in the upper or lower extremities [4]. The epidemiological and clinical characteristics of the infection often make it possible to distinguish the probable cause [2,3].

We reviewed the English-language literature using Pubmed and Scopus search databases on January, 2018 and select the main articles identified. The search terms browsed in the databases were “lymphocutaneous syndrome”, “nodular lymphangitis”, “sporotrichoid lymphocutaneous infections”, and “differential diagnosis”. We present a brief review of the articles found.

## 2. Sporotrichosis

The prototype of nodular lymphangitis is sporotrichosis [2]. *Sporothrix schenckii* is the most commonly recognized cause of nodular lymphangitis [12]. This thermodimorphic fungus grows in the soil and in the remains of plants and has been associated mainly with the thorns of the roses, corn cultivation and different types of plants. Cats are also considered a vector of the disease, which can emerge as a small epidemic in countries like Brazil where it is a public health problem [13]. Most cases have been reported in tropical and subtropical areas in the Western Hemisphere [14]. In the United States, sporotrichosis is most frequently seen in Oklahoma and in the Mississippi and Missouri river valleys [15]. The disease is also common in Mexico. Bonifaz et al. [16], have reported that most of the cases came from the states of Jalisco and Puebla with an incidence of 25 cases per 1000 inhabitants. One of the current problems in the management of sporotrichosis is the different susceptibility to antifungal agents, which is related to regional variations in the predominant subtypes [14,15].

Classical sporotrichosis characteristically manifests as a rapidly evolving lymphocutaneous infection (1 week to 3 months after the inoculum), which occurs after a traumatic injury [17,18]. Two classic cutaneous forms have been described: the fixed form constituted by a chancre without apparent lymphangitic dissemination, and the lymphatic form, which develops ulcerated nodules with lymphatic involvement (this last clinical variety is the most frequently seen) [17]. A nodule, often with a central ulceration that drains moderate amounts of seropurulent material, is typically formed at the site of the inoculum, which subsequently spreads to form more nodules following the lymphatic vessels. The course of the disease is usually asymptomatic, although it may present with fever and chills. Chronicity is common when it is not treated [18]. Other less frequent variants of sporotrichosis, representing less than 5%, however in a study published by Bonifaz et al. [19], a frequency of such variants represents 13.8%, include disseminated, visceral and fungemia forms, commonly associated with immunosuppression.

**Figure 1:**
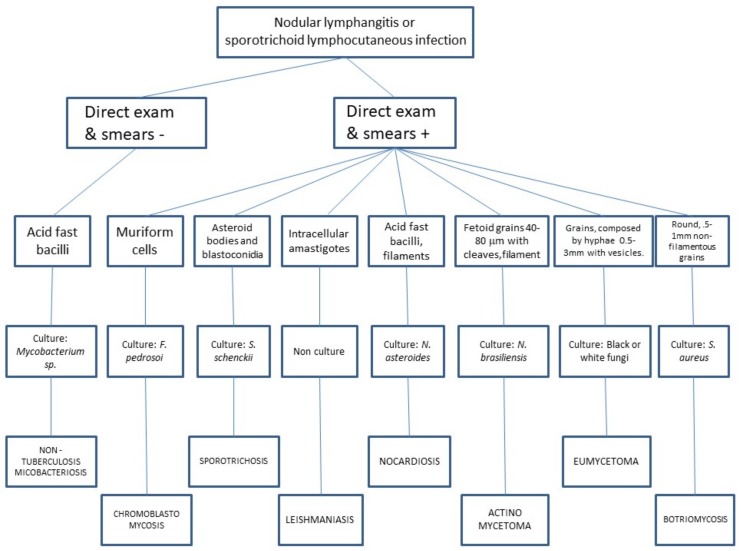
Algorithm proposal for diagnostic approach in nodular lymphangitis.

After recognizing the clinical syndrome in the appropriate epidemiological context, the diagnosis can be established by demonstrating the yeast in a biopsy sample using silver stains or by isolating the organism by culture (Figure 1). It is foreseeable that fungal forms are scarce or absent in tissue samples, and typically dispersed in the middle of a granulomatous inflammatory reaction [14]. Asteroid bodies are non-pathognomonic diagnostic clue before the conclusive demonstration of the etiological agent; this structure is frequently observed in histological sections of tissue infected with the fungus, which consists of a central extracellular yeast surrounded by bright eosinophilic spicules in a burst pattern within the microabscesses (similar to the Splendore-Hoeppli phenomenon in cases of botriomycosis) [20], or the observation of elongated yeast in the shape of a ship, more evident with PAS and Grocott stains.

Itraconazole is the treatment of choice for most patients with sporotrichosis [21], although, in our experience, treatment with potassium iodide is effective in cases of sporotrichosis with adequate cellular immune response (positive sporotrichin reaction) [18]. Terbinafine is also a potentially effective choice. Local heat applications (thermotherapy) or cryosurgery can accelerate resolution and can be considered as complementary therapies in specific situations, such as pregnancy or where there are potential interactions with other medications [22]. There are important variations in the results of antifungal susceptibility tests among the main subspecies of the *Sporothrix schenckii* complex, with *Sporothrix brasiliensis* being the most sensitive and *Sporothrix mexicana* the least [21]. The geographic distribution of susceptible subspecies in different regions of the world may affect the response to antifungal therapy. Terbinafine seems to be the most active agent in vitro, followed by ketoconazole, itraconazole, and posaconazole. Fluconazole and voriconazole are less active against *Sporothrix* strains [23].

Different laboratory methods can produce contradictory results and do not necessarily predict the clinical outcome. Regardless of the drug selection, treatment should be prolonged for more than 3 months, since the resolution of the infection is slow, and a relapse may occur despite the prolonged treatment [18].

## 3. Infections by *Mycobacterium marinum* and Other Mycobacteria

**Figure 2:**
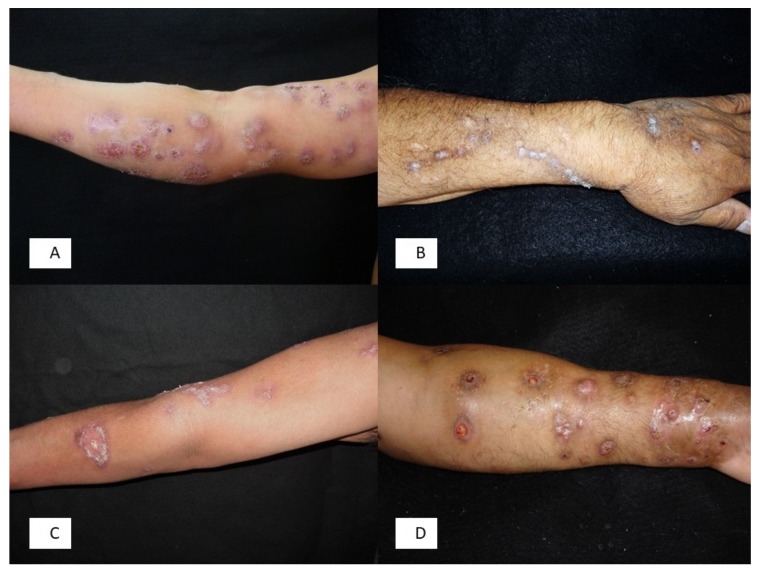
(**A**) Lymphatic sporotrichosis; (**B**) Chromoblastomycosis due to *Rhinocladiella aquaspersa*; (**C**) *Mycobacterium marinum* infection; (**D**) Actinomycetoma due to *Nocardia brasiliensis*.

Nontuberculous mycobacteria are an important cause of sporotrichoid lymphocutaneous infections. By far, the most prominently associated species with this clinical presentation is *Mycobacterium marinum*, a photochromic microorganism associated with fresh and salty water. Human infection occurs after injuries in aquariums or non-chlorinated swimming pools or through trauma from fish spines. After a variable incubation period that often exceeds 2 to 3 weeks, a slightly sensitive papule develops on the site of the inoculum, progressively enlarging and generating an ulcerative and suppurative lesion. Nodular lesions can develop following the direction of the lymphatic vessels, although they do not always reach the regional lymph nodes [24] (Figure 2).

The diagnosis of *M. marinum* infection is usually done by identifying the organism in the affected tissue by light microscopy and culture; the latter is the gold standard of diagnosis. Molecular studies such as Protein Chain Reaction (PCR) are not widely available for *M. marinum*. The cultures should be incubated at 32 °C [5].

Rifampin, ethambutol, trimethoprim-sulfamethoxazole and minocycline have been used successfully to treat this infection, often in two or three drug combinations. In our experience, minocycline is very effective even as monotherapy [24]. Other options include macrolides (particularly clarithromycin) and quinolones in treatment schedules administered for more than 3 months. The response is typically slow. The therapy is often maintained for 4 to 6 weeks after the lesions resolve [5].

*Mycobacterium chelonei* and *Mycobacterium fortuitum* are fast-growing mycobacteria that, similar to *M. marinum*, can develop nodular lymphangitis [2,12]. These organisms can be cultured in a period of 5 to 7 days in media for acid-fast bacilli with material obtainedfrom ulcerated areas or from debridement. The treatment of *M. chelonei* and *M. fortuitum* infection is less effective than the treatment for *M. marinum* infection, so it is advisable to determine the antimicrobial susceptibility before starting therapy. Clarithromycin and quinolones, which can be administered orally, and amikacin and cefoxitin, which require intravenous doses, have been effective in controlling and eradicating the infection [2,12]. Primary pulmonary pathogens (*Mycobacterium tuberculosis* and *Mycobacterium kansasii*) are rare causes of nodular lymphangitis that may develop as a result of spread of a distant focus or by direct inoculation [3].

The rare cases of sporotrichoid lymphocutaneous infections associated with *M. kansasii*, a photochromic acid, cosmopolitan bacterium, are characterized by asymptomatic verrucous plaques that appear at the site of the inoculum that occasionally follows a lymphatic path without affecting regional lymph nodes [3]. Sporotrichoid lesions due to *Mycobacterium haemophilum* infection have been described in a patient with an AIDS-related advanced-stage after trauma in an aquarium. Sporotrichoid lesions caused by *Mycobacterium avium-intracellulare* and *Mycobacterium flavescens* are rarely reported [3,12].

### 3.1. Nocardiosis

*Nocardia asteroides* and other *Nocardia* species can cause a subacute pyogenic disseminated infection that affects the lung, brain, and subcutaneous tissues in immunosuppressed patients, while in immunocompetent patients, it usually presents as a frequently localized cutaneous nocardiosis, manifested as a single painful abscess and/or an ulcerated papule, which appears a few days or weeks after a minor wound contaminated with the soil [25]. Patients infected with *Nocardia brasiliensis* (and less often *N. asteroides*) may develop lymphocutaneous sporotrichoid infections, sometimes with regional lymphadenopathy and mild systemic symptoms. The initial lesion can drain abundant purulent material, with the possibility of resolving without treatment. Up to 25% of patients with skin or soft tissue infections related to *N. brasiliensis* will develop sporotrichoid lesions [6,26].

*Nocardia* spp, are identifiable by Gram stain in wound drainage or in tissue samples, presenting as delicate ramifications of Gram-positive bacteria; most species are fast acid when stained with Ziehl–Nielsen stain. The diagnosis is confirmed by the isolation of the organism in routine culture media in a period ranging from 5 to 7 days [25]. The molecular identification is made by PCR amplification of 16S rRNA gene amplification and sequencing. As mentioned in previous lines, although the infection can be resolved slowly without treatment, short schemes with trimethoprim-sulfamethoxazole or minocycline can accelerate the resolution and prevent relapse.

### 3.2. Mycetoma

Mycetoma is a chronic, granulomatous infection of the skin that manifests as areas of swelling, nodules, or plaques with multiple fistulous trajectories, with drainage of purulent material with macroscopic granules of yellowish-white coloration [27]. It usually appears after a local trauma in the lower extremities, especially in the foot, followed by the trunk, back, and upper limbs. It is characterized by an infectious, suppurative process of the skin that can spread to the subcutaneous, muscular, and bone tissues, causing deformity of the affected area. It is caused by true fungi (black and white), mostly *Madurella mycetomatis*, and filamentous Gram positive bacteria, mostly *Nocardia brasiliensis*, followed by *Actinomadura madurae* [28]. All agents form grains, which are masses of filaments and these can be visible or not at a glance.

Mycetoma is a rare cause of nodular lymphangitis [29]. During the last decade, there has been an increasing incidence of mycetoma cases worldwide; this is probably due to a growing immunosuppressed population. The main predisposing factors include chronic lung disease, use of corticosteroids, diabetes mellitus, and malignancy, while the main factors that influence its dissemination include the transplantation of hematopoietic stem cells, leukemia, and diabetes mellitus. Once implanted, these organisms adapt to the environment and evade the host’s defenses. Soft tissue infections may be the initial presentation of the disease or a sign of hematogenous or, less often, lymphatic spread [30].

### 3.3. Leishmaniasis

Cutaneous leishmaniasis is endemic in rural areas of Central and South America. Cutaneous leishmaniasis occurs as a rural and urban disease in the Middle East and in large parts of Africa and tropical and subtropical Asia [31]. The parasites are transmitted by sand fly bites, living on the ground. After 2 to 24 weeks, a small nodule appears at the site of the inoculation that typically evolves into a shallow and well-defined ulcer [7,13].

Satellite lesions can be observed around the original lesion. Local pain is usually mild, unless complicated by bacterial superinfection. Regional lymphadenopathy and systemic symptoms are rare. The development of lymphocutaneous sporotrichoid infections in cutaneous leishmaniasis is frequent, especially in cases related to the *Leishmania brasiliensis* complex, mostly *Leishmania panamensis* or *Leishmania guyanensis*. Infections with the *Leishmania mexicana* complex are rarely related to nodular lymphangitis [7,13].

*Leishmania* amastigotes can usually be identified in material aspirated and stained with Giemsa or by scraping from the base of the ulcer. The natural history and the response to therapy are specific to each species [32]. Cutaneous leishmaniasis resolves spontaneously in most cases, but the healing process may require many months and leave a scarring area. Specific treatment in localized skin lesions is recommended for aesthetic purposes or when the causal species is related to potentially serious cases, such as mucocutaneous leishmaniasis [32,33].

Stibogluconate sodium or meglumine antimonite are effective options for systemic treatment; allopurinol has been successfully used as monotherapy. Certain azoles (mainly fluconazole and itraconazole) may be effective against some *Leishmania* species, such as *Leishmania major* and *Leishmania tropica* [33].

### 3.4. Tularemia

*Francisella tularensis* is a Gram-negative coccobacillus found throughout the United States, most commonly in Texas, Oklahoma, Arkansas, Tennessee, and Missouri [34]. Most patients acquire the disease from contact with infected mammals or from infected arthropod bites (e.g., ticks) [8]. The ulceroglandular form of tularemia occasionally includes sporotrichoid lesions. In some days after the inoculum, an ulcer of soft consistency develops, although the incubation period can be from 1 day to 2 weeks. The first lesions can be papulo-vesicular. Typical findings include severe constitutional symptoms and painful lymphadenopathy. The diagnosis is made by serological confirmation in a suggestive epidemiological environment [3]. Streptomycin is the drug of choice for tularemia; relapses are more frequent after treatment with tetracyclines [8].

### 3.5. Bacillus

*Bacillus anthracis* can also cause nodular lymphangitis: a pruritic papule that progresses rapidly to ulceration and necrosis after exposure to cattle, sheep or goats or their skins is characteristic of the cutaneous form of anthrax [9,35]. Local lymphangitis and lymphadenopathy occur, and systemic symptoms may progress. Gram-positive bacilli are easily identified in the smear, and cultures should be handled with caution by laboratory professionals [36]. Careful handling of industrial products has reduced the risk of cutaneous anthrax. Treatment with penicillin, erythromycin, or tetracycline is effective in reducing the severity of systemic symptoms and mortality rates [37].

There have been infrequent reports of nodular lymphangitis or sporotrichoid lymphocutaneous infections by organisms other than those already mentioned. In contrast to patients who develop a disseminated disease, patients with localized skin infections are usually immunocompetent and rarely develop sporotrichoid lymphocutaneous infections [3].

Other rare organisms associated with nodular lymphangitis include *Staphylococcus aureus* [10], *Streptococcus pyogenes*, and *Pseudomonas pseudomallei*, although the only pyogenic bacteria frequently documented in association with sporotrichoid lesions are group A *Streptococci* and coagulase-positive *Staphylococci* [1]. Despite being a great simulator, no convincing cases of sporotrichoid lesions associated with syphilis have been reported. A variety of fungi other than *Sporothrix*, including *Coccidioides immitis/Coccidiodes posadasii*, *Blastomyces dermatitidis*, *Histoplasma capsulatum*, *Cryptococcus neoformans*, *Scedosporium* species, and some agents of chromoblastomycosis (*Rhinocladiella aquaspersa)* [38] have been associated with sporotrichoid lesions. Many of these endemic fungal infections occur in laboratory professionals. Viral causes associated with sporotrichoid lesions are rare, with the herpetic whitlow being the most common. A case of sporotrichoid lesions caused by vaccinia virus (cowpox) has been described [2,11,14].

## 4. Clues to Differential Diagnosis

Cutaneous infections caused by *S. schenckii*, *L. panamensis*, *L. guyanensis*, *N. brasiliensis*, *M. marinum,* and *F. tularensis* are the most likely ones to be complicated by sporotrichoid lesions, commonly occurring in immunocompetent hosts and remaining confined to lymphocutaneous structures (Table 1). In an immunocompromised host, the possibility that a skin lesion represents a hidden spread of infection should always be carefully considered and ruled out [2,12,14].

By prioritizing the causes of nodular lymphangitis in a particular patient, the epidemiological context in which the infection develops is a useful but not definitive discrimination factor. Infections subsequent to injuries suffered during gardening are not caused exclusively by *Sporothrix* sp. Sporotrichoid lesions developed after a trivial traumatic wound, contaminated with soil or water, may be due to nocardiosis or infection by rapidly growing mycobacteria and not necessarily sporotrichosis [3,12].

Infection of a laceration in a marine environment or a recent wound exposed to salty water increases the possibility that *M. marinum* is the causative agent. Nodular lymphangitis in a hunter involves *F. tularensis*, as does an infection after a deer fly bite or exposure to ticks. Pathologists and microbiologists who work with certain fungi can develop skin infections that are rarely complicated by sporotricidal lesions after accidents in the laboratory. Geography should also be taken into account when formulating an individualized list of diagnostic possibilities (Table 2). For example, tularemia is confined to the northern hemisphere, while leishmaniasis is unlikely to be acquired in the north of Texas. The associated clinical circumstances and an estimated incubation period can provide additional information useful for the etiological diagnosis. Infections secondary to *F. tularensis*, *S. pyogenes*, *S. aureus,* and *Pseudomonas pseudomallei* have the shortest incubation periods among the agents causing sporotrichoid lesions (typically less than a week). Tularemia is the only common cause of sporotrichoid lesions characterized by an incubation period measured in days, in association with fever, systemic symptoms, and severe lymphadenitis. Nocardia infections may also have a relatively short incubation period. The time between the inoculum and the development of the disease is approximately 1 to 2 weeks for infections caused by fungal species and mycobacteria [4,12,13].

The skin lesions observed in sporotrichosis, leishmaniasis, nocardiosis, and tularemia caninitially develop as papules or nodules that typically ulcerate. In cutaneous mycobacteriosis, the primary focus tends to be nodular. Only *F. tularensis* commonly causes excruciatingly painful ulcers, which explains its description as “chancriform”. In addition to tularemia and nocardiosis, none of the common causes of sporotrichoid lesions are associated with pain at the inoculum site. Nocardia lesions may exude frank purulent material, while mycobacterial lesions usually have a more sparse seropurulent drainage. Multiple satellite lesions can be seen with these organisms. Lymphangitic nodules can sometimes progress to suppurative ulcers, particularly in *Nocardia* infections. *F. tularensis* prominently involves regional lymph nodes, as implied by the designation “ulcero-glandular tularemia”; the associated lymphadenitis is usually painful and may overshadow the primary skin lesion. Regional lymphadenopathy develops less frequently in patients infected with *N. brasiliensis* and is unusual in *Sporothrix* or *M. marinum* infections. Leishmaniasis can occasionally be associated with a moderate and non-painful enlargement of a solitary regional lymph node [2,11,14].

The lack of response to a specific therapy can sometimes provide diagnostic clues, but should be interpreted with caution. Patients with sporotrichoid lesions are often treated empirically as if they had sporotrichosis without a definitive diagnosis. In this context, the lack of response after a prolonged therapeutic trial should encourage the consideration of other causes, prioritized according to the clinical presentation and the epidemiological context. Unfortunately, many patients with sporotrichoid lesions respond slowly even to optimal therapy. Apparent responses can be misleading because spontaneous recovery can occur in many of these infections, without being administered a specific therapy [2,12,13,14].

## 5. Conclusions

Most cases of nodular lymphangitis or sporotrichoid lesions are the result of infectious and noninfectious diseases. Skin lesions caused by sporotrichosis, leishmaniasis, nocardiosis, mycobacteriosis, and tularemia are the most likely infections that are complicated by nodular lymphangitis or sporotrichoid lesions. A detailed clinical history (emphasizing the triggering lesion, the epidemiological context, the probable incubation period, the rate of progression, and the presence or absence of constitutional symptoms) and a careful physical examination (focusing on the appearance of the primary skin lesions and regional lymph nodes) with the help of cultures, histopathology, and molecular studies (PCR), will allow the initiation of a specific therapy in the majority of patients. Antimicrobial treatment without surgical intervention is usually sufficient, although recovery should be considered slow.

## Figures and Tables

**Table 1:** Infectious causes of nodular lymphangitis.

*Sporothrix schenckii*
*Blastomyces dermatitidis*
*Coccidioides immitis*
*Histoplasma capsulatum*
*Cryptococcus neoformans*
*Scedosporium apiospermum*
*Fusarium sp.*
*Scopulariopsis blochii*
*Nocardia sp.* *(N. brasiliensis, N. asteroides, N. otitidiscaviarum (caviae), N. transvalensis)*
*Pseudomonas (Burkholderia) pseudomallei*
*Francisella tularensis*
*Staphylococcus aureus*
*Streptococcus pyogenes*
*Bacillus anthracis*
*Mycobacterium sp.* *(M. marinum, M. chelonae, M. kansasii, M. avium-intracellulare, M. tuberculosis, M. fortuitum, M. flavescens, M. abscessus, M. haemophilum)*
*Leishmania (viannia) sp.* *(L. brasiliensis (guyanensis/panamensis), L. tropica, L. major)*
*Cowpox virus (Vaccinia virus)*
*Herpes simplex*
*Erysipelothrix rhusiopathiae*

**Table 2:** Characteristics of the main causes of nodular lymphangitis.

Agent	Geographical Distribution	Risk Factors (Sources)	Incubation Period	Primary Lesion	Pathology/Culture	Treatment
*Sporothrix schenckii* (complex) [2]	Tropical and subtropical América	Gardening (roses), corn crop, soil contact, sphagnum moss, animal scratches (cats) or bites.	1 week–3 months	Painless ulcerated nodule.	Granulomatous infiltrate with asteroids bodies and elongated yeast/Culture (Sabouraud agar at 32 °C)	Itraconazole 200 mg/daily; SSKI 5 drops in water 3 times daily, increasing slowly to 40–50 drops 3 times daily as tolerated; terbinafine 250 mg bid. Duration: 2 months after the resolution of lesions
*Mycobacterium marinum* [5]	Worldwide	Aquariums, fish-handling (fresh and saltwater fish), swimming in oceans, lakes, pools.	1–6 weeks	Mildly tender, often ulcerated nodule, with scant seropurulent exudate.	Suppurative granulomas/Culture on Lowenstein Jensen or Middlebrok agar at 30–32 °C.	Rifampin (15 mg/kg qd) + ethambutol (25 mg/kg qd); minocycline 100 mg bid; Rifampin (same dose) + clarithromycin (30 mg/kg qd); duration: 2–3 months after resolution of symptoms.
*Nocardia brasiliensis/N. asteroids* [6]	Worldwide	Soil exposure, botanicals, cat scratch.	3 days–6 weeks	Tender nodules commonly ulcerated with mild to abundant purulent drainage.	Granulomas and sulphur granules, giant cells, abscess formation/Culture in most media.	TMP-SMX 160/800 mg tid; minocycline 200 mg bid. Duration: 3 months. Amikacin, surgical excision are also effective options.
*Leishmania brasiliensis* [7]	Central and South America	Residence in or travel to endemic areas.	2–24 weeks	Painless, well-demarcated shallow ulcer with indurated borders.	Amastigotes within histiocytes/Culture on tissue biopsy or impression smears usually in Nicolle-Novy-Macneal medium or animals.	Stibogluconatesodium 20 mg/kg qd or meglumine antimonite 20 mg/kg qd; amphotericin B 0.25–1 mg/kg qd. Duration 20 days. Antimonials, itraconazole, ketoconazole and allopurinol are also effective options.
*Francisella tularensis* [8]	Northern hemisphere	Hunting and other rural outdoor activities, transmitted through ticks, deer flies, wild mammals (rabbits, squirrels, voles), cats.	1–6 days	Painful ulcerated papule with suppuration.	Granulomatous reaction, multinucleated giant cells, epithelioid cells, neutrophils with focal necrosis/Serology, culture isolation with safety hood.	Streptomycin, tetracycline and gentamicin are useful treatment options.
*Bacillus anthracis* [9]	Worldwide	Exposure to infected animals, animal products, or spores in the soil.		Painless ulcer with vesicles, edema.	Gram stain and culture; serologic testing and punch biopsy at the edge of the lesion, examined by silver staining and immunohistochemical testing.	Penicillin G, ciprofloxacin and doxycycline.
*Staphylococcus aureus* [10]	Worldwide		4–10 days	Nodular lesions, frequently abscessed, with drainage of purulent fluid.	Botryomycotic grains/Culture on most media.	Antibiotics mainly beta lactams (Cefazolin, Cefadroxil).
*Coccidioides immitis/C. posadasii* [11]	Southwest USA and Northern Mexico	Soil, laboratory contamination, thorns.	1–4 weeks	Verrucous plaques and ulcerated nodules.	Chronic granulomatous infiltrate with plasma cells and spherules/Culture on Sabouraud agar at 32 °C.	Itraconazole, fluconazole, amphotericin B.
*Blastomyces dermatitidis* [11]	Worldwide usually North America	Soil, cat scratches, dog bites, laboratory contamination.	1–5 weeks	Verrucous plaques and ulcerated nodules.	Chronic granulomatous infiltrate with plasma cells/Culture on Sabouraud agar at 32 °C.	Itraconazole, fluconazole, amphotericin B.
*Histoplasma capsulatum* [11]	Worldwide	Soil, birds, chickens, laboratory contamination.	5–18 days	Umbilicated papules, ulcerated nodules with necrosis, vesicles (rash herpetiformis), pustules, acneiform rashes, verrucous plaques, psoriasis-like papulosquamous lesions and purpura.	Chronic granulomatous infiltrate with plasma cells/Culture on Sabouraud agar at 32 °C.	Itraconazole, fluconazole, amphotericin B.
*Scedosporium* sp. [11]	Worldwide	Soil, water, sewage (immunosuppresion)	Unknown	Partly suppurating nodules.	Chronic granulomatous infiltrate/Culture on Sabouraud agar at room temperature.	Itraconazole and ketoconazole.
*Herpes simplex* [11]	Worldwide		2–12 days	Vesicles distributed on a linear fashion.	Multinucleated giant cells with intranuclear inclusion bodies / tissue cell culture, Tzanksmear and serology.	Acyclovir, famciclovir and valacyclovir. Foscarnet.
*Cowpox virus* [11]		Cattle, cats.	Unknown		Unspecific histology/electron microscopy (brick-shaped virus using tungstic acid-stained native material) and serology.	Supportive

SSKI = Saturated Solution Potassium Iodide; bid = twice daily; qd = once daily; tid = three times daily.
